# Six Cases of Abdominal Section

**Published:** 1892-07

**Authors:** M. A. Crockett

**Affiliations:** Assistant Gynecologist to the Buffalo General Hospital; Gynecologist to Fitch Dispensary; Instructor in Obstetrics and Gynecology, Medical Department, University of Buffalo


### SIX CASES OF ABDOMINAL SECTION.[1]

BY M. A. CROCKETT, A. B., M. D.,

Assistant Gynecologist to the Buffalo General Hospital; Gynecologist to Fitch Dispensary; Instructor in Obstetrics and Gynecology, Medical Department, University of Buffalo.

IN THESE days, when laparatomists count their operations by the hundred, it seems rather presumptuous for me to present a report upon a paltry half dozen cases. In connection with some of these patients, however, there are a number of interesting features which, I trust, will justify me in asking your attention for a short time this evening.

I have been fortunate enough to have no deaths thus far. In two or three cases, stitch-hole abscesses occurred; otherwise the recoveries were uneventful.

Numbers one, three, and six were ordinary cases of chronic salpingitis and ovaritis. Each of these patients had been ill for from two to five years, and had received treatment from a number of physicians. They belonged to the class commonly designated as "wrecks," and despaired of ever obtaining relief from their sufferings. The operations were of ordinary difficulty, the adhesions being numerous in each case, and the specimens removed fully justifying the treatment. The tubes and ovaries presented the lesions of chronic inflammation, *e. g.*, closure of fimbriated ends, hydrosalpinx, cirrhosis, and cystic degeneration of the ovaries, rendering these organs physiologically useless and sources of misery to their possessors.

As regards the ultimate results, it is, unfortunately, rarely possible to follow up hospital cases. It is also the fact that the benefits of operation are not always immediate. Number one had improved greatly before leaving the hospital. The second patient was in good health six months after operation. The third patient has not yet been discharged, but is doing well in every respect.

It is not the place here to discuss the question of removal of the uterine appendages. The subject, periodically, occupies considerable space in our journals, and too often is the cause of exhibitions of bitterness and prejudice by no means creditable to the profession.

I would only ask you to consider each case on its individual merits. "It is a condition and not a theory which confronts us." Very often the patient has to choose between operation and the almshouse.

1. Read before the Buffalo Clinical Society, May 14, 1892.

How useless, in such cases, to talk about treatment by electricity, local applications, massage, tonics, rest, etc., when such treatment cannot be obtained by our patient. By operation these women hope to return to health by a short cut. Unfortunately, sometimes this short cut turns out to be a rough and dangerous path, and may even prove to be no thoroughfare at all. On the other hand, in many cases operation results in a rapid and complete cure. Let us, therefore, try to be fair to an operator, and not condemn until we know the facts.

Case number two is a rather remarkable recovery from a severe operation. The history is as follows :

Age, twenty-five ; married one year ; never pregnant. Clear history of gonorrheal infection previous to marriage. Brought to the hospital flowing profusely, and suffering from severe abdominal pain. Patient attributed her trouble to exposure to cold during her last menstrual period.

Vaginal examination showed a retroverted uterus, with large fluctuating masses on either side. After a few days the temperature presented a typical pus curve, and a diagnosis of pyosalpinx, probably gonorrheal, was made. Operation August 29, 1891, Dr. Parmenter assisting. The adhesions were numerous and very vascular. The right tube and ovary had to be torn loose from vermiform appendix, small intestines, floor of pelvis, etc. The ovary was converted into an abscess the size of a hen's egg. The tube resembled a small sweet potato, and contained several ounces of pus.

On the left side everything was buried in adhesions. Attempts to free the tube resulted in tearing off small pieces ; the consistency being like that of wet blotting paper. Everything in the pelvis was floating in pus, and the condition of the patient became extremely bad. A considerable portion of the tube remained behind in the bottom of the pelvis. The pelvis was sponged out and the abdomen irrigated. At Dr. Parmenter's suggestion, the abdomen was left half full of hot water. This procedure had a very happy effect upon the pulse, and the patient began to rally at once. Finally, a glass drainage tube was inserted, and the wound closed.

This temperature chart shows you the condition of the patient before and after operation. You will notice that for a week before operation her temperature at evening varied between 101° and 103.4°. During the first eighteen days subsequent to operation her temperature did not touch 100°, except on the eighth day, when a stitch-hole abscess caused a temporary rise. The temperature fell again upon evacuation of the pus. Considerable pus discharged through the drainage tube, which remained in place for forty-eight hours.

I was much surprised at the toleration shown by the peritoneum in this case. This pus caused marked constitutional disturbance before operation, and yet no bad result followed its escape from the tube. The discharge from the drainage tube showed that the pelvis was not clean after operation. The recent investigations of Wertheim (*Archiv. für Gyn.*, Bd. XLII.), show that the gonococcus alone can produce pus, and that mixed infection is not necessary in order to explain gonorrheal pyosalpinx. He also shows that the gonococcus affects the peritoneum only to a very limited extent. I think these ideas afford some explanation of the favorable course of this case. When the patient left the hospital, no evidence of any pelvic trouble could be found on vaginal examination.

Case number four shows how difficult it is, sometimes, to diagnosticate intra-abdominal lesions. I first saw the patient in September, 1891, and obtained the following history:

Age, seventeen; single. Last menstrual period, ten weeks ago. Five weeks ago she noticed that her abdomen was increasing in size. At times, usually at night, she had severe pains in this "swelling." The patient was very anemic-looking, and her face drawn and haggard. The enlargement of the abdomen was very marked. Palpation disclosed a smooth, hard, rounded tumor, reaching almost to the level of the umbilicus. The tumor was movable, and not at all sensitive, unless deep pressure was made slightly above the left groin.

Two fingers could easily be introduced into the vagina. The cervix was low down, and the tumor could be felt on all sides. I concluded that I had to do with a uterine tumor, but was not at all clear as to its nature. The patient was sent to the hospital ward of the Ingleside Home, under the care of Dr. Lillian C. Randall. The patient's temperature was taken a number of times, and on each occasion found to be normal.

Later, Dr. Mann saw the case. His opinion was that the tumor was sarcomatous, and as the general condition was then very bad, I gave an unfavorable prognosis to the family.

About a week later, I was informed that the tumor had suddenly disappeared, and an examination showed that certainly three-quarters of the mass had vanished. There remained, however, a distinct tumor on the left side of the uterus, which now could easily be made out. The patient's general health rapidly improved, and six weeks later she went home. No further change in the local conditions had occurred. The patient had not menstruated during her stay in Buffalo.

About one month later the patient returned, complaining of severe pains in the left side. She was absolutely unable to do any work, and desired an operation.

Examination showed a firm, rounded tumor, the size of the closed fist, lying on the left side of the uterus. Patient had had a normal menstrual period while at home.

Operation January 29, 1892, Dr. Mann kindly helping me. The mass on the left side was easily reached. Its surface was perfectly smooth, as though it were covered by the distended broad ligament. On cutting through this covering with the idea of enucleation, it was found to consist of almost half an inch of inflammatory tissue. While working my way downward there was a sudden gush of vile smelling pus, and from the midst of this tissue this tube and wreck of an ovary was dug out.

The right tube and ovary were adherent, and showed signs of permanent pathological changes. They were, therefore, removed, drainage tube inserted, and abdomen closed. The subsequent history is of no especial interest. The patient left the hospital free from pain, and feeling quite well.

I have here the appendages from this case. The left ovary is represented by the remains of this sac. The tube contained a small amount of pus near the fimbriated end. I cannot tell whether I ruptured the pus sac during removal, or whether the pus had already escaped ; the other ovary, as you see, is very large. The end of the tube was closed, but it contained no pus.

I presume the large mass originally present was entirely inflammatory. The case was seen by a number of physicians, and no one suspected for a moment the true state of affairs. The rapid disappearance is attested by several reliable observers, but the fact has never been satisfactorily explained. The patient's subsequent history is uninteresting. She was quite well in every respect when discharged from the hospital.

The last case which I wish to report (number five) is one of extra-uterine pregnancy. The specimen, which I show you, is certainly very perfect.

Patient aged twenty-seven ; married eight years ; two children, the last born five years ago ; no abortions. Menstruation has always been rather profuse, and sometimes occurred at greater intervals than twenty-eight days. The patient has had good health, and never complained of any local or general disturbances. She menstruated December 14, 1891, but not at all in January, 1892, and, therefore, believed herself to be pregnant. About the middle of February she was seized with a sudden and severe attack of cramps in the abdomen, and, as a result, was confined to her bed for over a week. On attempting to get up and attend to the house, the abdominal pains returned, and from this time on she was obliged to remain in bed. About the first of April she was seen by Dr. Mann, who made a diagnosis of probable extra-uterine pregnancy, with rupture of the sac. Soon after she entered the Buffalo General Hospital for treatment. Owing to Dr. Mann's illness the case was turned over to me. I examined her about the latter part of April, and found the following conditions present:

A large mass could be felt to the right of the uterus, displacing that organ towards the left. Bimanual examination showed the tumor to be about the size of a cocoanut. It rose high enough in the pelvis to be felt by the outside hand alone.

Operation was performed May 2, 1892, Dr. Parmenter assisting.

A number of coils of small intestines had to be separated from the top of the mass before the hand could be introduced. An extensive raw surface was left upon the gut, and in one place sutures through the peritoneal coat were deemed necessary. The right tube merged into the tumor and was tied off. By slowly working down under the tumor with the fingers, the mass was removed entire, a large area of raw surface being left behind, and for a time the oozing was considerable. The patient being in Trendelenburg's position, I was enabled to secure the largest vessel, and pressure with sponges soon checked all further bleeding. The abdomen was closed without drainage.

Examination of the specimen shows that it had evidently ruptured from the tube into the broad ligament, from which it was enucleated. The bulk is more or less organized fibrine. I have laid open the amniotic sac, which shows plainly. The fetus is about three and a half months old, and is hanging by the cord from the placenta. I will pass around the temperature chart. Today is the thirteenth day, and the temperature has never been above 100°, except one evening. Considering the extensive raw surface necessarily remaining in the abdomen, the temperature curve is rather remarkable. The wound has united by first intention, and I shall allow the patient to sit up tomorrow.

---

Deaths from Influenza.—Dr. Lewis A. Balch, the Secretary of the State Board of Health, estimates that 10,000 deaths in the State of New York were chargeable to influenza and its sequelæ in the Winter quarter of 1892. The local diseases appear to bear the brunt of a largely increased mortality, but influenza is, probably, the genuine cause.

